# Incidence of adverse cardiovascular events associated with immune checkpoint inhibitors and risk factors for left ventricular dysfunction: A single-center prospective clinical study

**DOI:** 10.3389/fcvm.2023.1052699

**Published:** 2023-01-23

**Authors:** Chuan Zhang, Zhulu Chen, Shu Qin, Yuxi Zhu, Linjie Shu, Zhong Zuo

**Affiliations:** ^1^Department of Cardiology, The First Affiliated Hospital of Chongqing Medical University, Chongqing, China; ^2^Department of Oncology, The First Affiliated Hospital of Chongqing Medical University, Chongqing, China

**Keywords:** immune checkpoint inhibitor, cardiotoxicity, left ventricular dysfunction, myocarditis, myocardial fibrosis

## Abstract

**Background:**

The incidence of immune checkpoint inhibitors (ICI)-related adverse cardiovascular events (ACEs) may be underestimated, and there are few reports on the incidence and risk factors of ICI-induced left ventricular dysfunction (LVD).

**Objectives:**

This study aimed to investigate the incidence of ACEs caused by ICI, in particular to analyze the incidence and risk factors of LV systolic and diastolic dysfunction.

**Materials and methods:**

A prospective clinical study was performed on patients who received ICI in our hospital from November 2020 to October 2021. They received regular cardiovascular examinations, including echocardiography, ECG, cTnT, and NT-proBNP, etc. The incidence of various ACEs was counted, and the risk factors of LVD were analyzed.

**Results:**

A total of 106 cancer patients treated with ICI were recruited. During the follow-up, 41 patients (38.68%) developed various ECG abnormalities, 39 patients (36.79%) developed LVDD, 9 patients (8.49%) developed CTRCD, and 2 patients (1.89%) developed new pericardial effusion. The patients with elevated cTnT, CK-MB, and NT-proBNP were 10 (9.43%), 8 (7.55%), and 8 (7.5%), respectively. Thirteen of the 52 patients with LVD had hypertension, while 4 of the 54 patients without LVD had hypertension (OR = 4.17, 95% CI: 1.26–13.78; *P* = 0.019). The baseline LVEF and LVFS of patients with LVD were 61.54 ± 4.15% and 33.78 ± 2.73%, while those of the control group were 64.16 ± 3.68% and 34.95 ± 2.84, respectively (*P* = 0.003 and *P* = 0.048). Compared with patients without LVD, patients with LVD had lower e’ (6.99 ± 1.33 cm/s vs. 7.64 ± 1.39 cm/s, *P* = 0.029) and higher E to e’ ratio (11.89 ± 3.15 cm/s vs. 10.43 ± 2.52, *P* = 0.024). Multiple regression analysis showed that a history of hypertension (HR = 26.52, 95% CI: 2.479–283.667, *P* = 0.007) and lower baseline e’ (HR = 0.04, 95% CI: 0.003–0.709, *P* = 0.028) were risk factors for developing LVD.

**Conclusion:**

Patients treated with ICI may develop multiple ACEs, including acute myocarditis, pericarditis, ECG abnormalities, and elevated cardiac biomarkers. ICI may lead to a high incidence of LVD, and echocardiography is helpful for early detection of LVD. Patients with hypertension or poor LV systolic or diastolic function at baseline were predictors of LVD after ICI treatment.

## Background

Immune checkpoints are a series of cellular receptors expressed on the cell surface that play a role in negative immune regulation. They play an important role in maintaining immune system balance and preventing autoimmunity (1). Currently, cytotoxic T lymphocyte-associated antigen 4 (CTLA-4), programmed cell death 1 (PD-1) and programmed cell death ligand 1 (PD-L1) are the most studied immune checkpoints. Immune checkpoint inhibitors (ICIs) are specific monoclonal antibodies against these immune checkpoints, which can activate the immune system to kill tumor cells (2). In the past 10 years, various ICIs have been gradually applied in clinical practice and have become a new progress in the field of tumor therapy (3, 4). However, activation of the immune system by ICI may lead to immune damage to one’s own tissues or organs. In recent years, there have been numerous reports of immune-related adverse events (irAEs) in cancer patients treated with ICI, some of which were fatal (5, 6). Among them, although the incidence of adverse cardiovascular events (ACEs) caused by ICI is low, serious consequences and even death have occurred in some patients. In the past few years, reports of fulminant myocarditis or fatal heart failure caused by ICI have attracted the attention of the medical community (7–9).

However, ICI have only been used clinically for a few years and there is limited understanding of their cardiotoxicity. Current studies on ICI-related cardiotoxicity are mostly case reports, database-based statistical analyses, or retrospective studies, and the incidence of ACEs may be underestimated. On the other hand, most patients treated with ICI do not undergo regular echocardiography, resulting in limited data on left ventricular dysfunction (LVD), and the risk factors are poorly understood. LVD includes LV systolic and diastolic dysfunction. LV systolic dysfunction is one of the major cardiovascular complications associated with oncology drugs (10), known as cancer therapy-related cardiac dysfunction (CTRCD). LV diastolic dysfunction (LVDD) is associated with both cardiovascular and non-cardiovascular mortality (11), and cancer treatment may also contribute to LVDD (12). A study suggested that the development of LVDD in cancer patients may be significantly associated with the development of systolic dysfunction and all-cause mortality (13). However, studies on the incidence of LVDD caused by ICI are currently lacking.

This study is a prospective clinical study, and the purpose of our study is to regularly monitor the occurrence of cardiotoxicity in patients treated with ICI by electrocardiography (ECG), echocardiography, cardiac biomarkers, etc. In particular, echocardiography is used to detect LVD, including CTRCD and LVDD, and then we will count their incidence and screen for clinical predictors of LVD development through statistical analysis.

## Materials and methods

### Study population

This prospective study included all cancer patients who received ICI at the First Affiliated Hospital of Chongqing Medical University from November 1, 2020 to October 31, 2021. Members of our research team assessed the clinical status of these patients and interviewed patients who met the inclusion criteria. Patients were included in the study if they agreed to undergo periodic examinations, such as ECG, cardiac biomarkers, and echocardiography, as well as clinical follow-up if necessary.

#### Inclusion criteria

(1) The patient received at least 2 cycles of ICI treatment; (2) Patients agreed to undergo at least one ECG, cardiac biomarker, and echocardiogram before and after treatment; (3) Before receiving ICI, the patient’s cardiac function was assessed as grade 3 or less by NYHA criteria. If the patient underwent echocardiography, the left ventricular ejection fraction (LVEF) should be greater than or equal to 50%.

#### Exclusion criteria

(1) Received known cardiovascular toxicity drugs, such as anthracyclines, HER-2 targeted drugs, etc., at the same time or within 3 months of ICI treatment; (2) Before receiving ICI, the patient’s cardiac function was assessed as grade 4 according to NYHA criteria, or there was a history of myocardial infarction or acute heart failure within 3 months, or the LVEF by echocardiography was less than 50%; (3) Clinically diagnosed with acute viral myocarditis or infective endocarditis within 30 days; (4) Severe liver or renal insufficiency; (5) Severe pneumonia or respiratory failure; (6) A history of cardiomyopathy such as dilated cardiomyopathy, alcoholic cardiomyopathy, and myocardial amyloidosis; (7) The electrocardiogram showed atrial flutter or atrial fibrillation.

### Basic information

Patients included in the study were asked to provide a detailed medical history and to undergo a physical examination after admission. Basic information is recorded in detail, including the patient’s clinical diagnosis: type and stage of cancer, and whether surgery was performed; demographic characteristics, including the patient’s sex, age, height, weight, nicotine/tobacco use history, and alcohol intake; history of cardiovascular disease and risk factors; details of use of conventional chemotherapy drugs or ICI. ECG, chest computer tomography (CT), and cardiac biomarkers including cardiac troponin T(cTnT) and N-terminal pro brain natriuretic peptide (NT-proBNP) were performed on patients before and during treatment with ICI, and the results were recorded in detail.

### Cardiac function assessed by echocardiography

Echocardiography was performed in accordance with the 2019 American Society of Echocardiography (ASE) guidelines for adult echocardiography (14). The examination items include the diameter of the heart chambers, the systolic and diastolic function of the LV. The evaluation indexes of LV systolic function were LVEF and left ventricular fraction shortening (LVFS), and LVEF was measured by biplane Simpson method.

The evaluation indexes of LV diastolic function were peak early diastolic mitral inflow velocity (E), peak early diastolic mitral annulus velocity (e’) and the ratio of the two (E/e’) (15). E was obtained from color flow imaging of the mitral valve obtained by pulsed-wave doppler imaging. e’ (septal and LV lateral wall) was measured by tissue doppler imaging (TDI). E/e’ is calculated by dividing the mitral valve E velocity by the mitral annular e’ velocity.

### Study endpoints

1) ICI-associated myocarditis: (1) Endomyocardial biopsy or autopsy with typical histological features of myocarditis; or (2) Guidelines-recommended scoring system for clinically suspected myocarditis, including clinical, biomarker, and imaging features (16).

2) New abnormal ECG, abnormal cardiac biomarkers, pericardial effusion. Among them, abnormal biomarkers were defined as cTnT > 0.030 μg/L or NT-proBNP > 300 ng/L.

3) CTRCD: LVEF decreased by ≥ 10% from baseline and absolute value < 50% (17).

4) LVDD: Meet the following two. (1) E/e’ > 14. (2) At the level of the LV mitral annulus, e’ < 7 cm/s on the septal side or e’ < 10 cm/s on the LV lateral wall (15).

### Statistical analysis

Continuous variables such as age, weight, etc., were described as mean ± standard deviation, and categorical variables such as gender, history of hypertension, etc., were described as percentages. According to pre-defined criteria, the number of patients with LVD (including CTRCD and LVDD) after receiving ICI was counted, and the percentage was calculated. Pericardial effusion, abnormal ECG and abnormal cardiac biomarkers were counted after ICI treatment, and results are presented as number of cases and percentages. The average value of LV cardiac function before and after ICI treatment in cancer patients was counted.

Statistical analysis was performed using SPSS 21.0 statistical software (IBM, Armonk, New York, USA). The K-S test was used to assess its normality. Continuous variables were compared using Student’s *t*-test or Wilcoxon rank-sum test based on their normality. One-way ANOVA was used to compare the means of 3 groups or more. Univariate and multivariate regression were used to analyze risk factors for LVDD. All *P*-values are two-sided, with *P* < 0.05 considered statistically significant.

### Ethical approval and informed consent

This study was conducted in strict accordance with the requirements of the Declaration of Helsinki. Our research also passed the ethics review by the Ethics Committee of The First Affiliated Hospital of Chongqing Medical University (ethics number, 2018-10-2).

## Results

### Basic characteristics

#### Demographic characteristics

A total of 106 cancer patients treated with ICI were included in this study, and the mean follow-up time was 4.7 months. The average age was 60.08 ± 8.47 years (35–76 years), and the proportion of males was 85.8% (91/106). The mean body weight was 60.95 ± 9.48 kg, the mean BMI was 22.56 ± 2.93, and 17 patients had a BMI greater than 25. The mean systolic blood pressure (SBP) of the included patients was 124.01 ± 16.25 mmHg and diastolic blood pressure (DBP) was 76.94 ± 9.37 mmHg. According to the diagnostic criteria for elevated blood pressure (SBP ≥ 140 mmHg or DBP ≥ 90 mmHg), 26 patients had elevated SBP and 4 patients had elevated DBP. The average heart rate was 83.35 ± 10.22 beats/min, more than 100 beats/min in 5 people, and less than 60 beats/min in 1 person (Table 1).

**TABLE 1:** Baseline characteristics of patients and univariate regression analysis between studied parameters and left ventricle (LV) dysfunction.

	Total (*n* = 106)	Normal (*n* = 54)	LV dysfunction (*n* = 52)	Unadjusted OR (95% CI)	*P*-value
**Demographic characteristics**					
Sex (male)	91 (85.8)	46 (85.2)	45 (86.5)	1.12 (0.37–3.34)	0.842
Age (years)	60.08 ± 8.47	59.04 ± 9.41	61.17 ± 7.31	1.03 (0.98–1.08)	0.196
Wight (kg)	60.95 ± 9.48	60.94 ± 9.95	60.95 ± 8.97	1.00 (0.96–1.04)	0.997
BMI	22.56 ± 2.93	22.68 ± 2.98	22.44 ± 2.90	0.97 (0.85–1.11)	0.678
HR (bpm)	83.35 ± 10.22	84.28 ± 10.87	82.38 ± 9.51	0.98 (0.94–1.02)	0.343
SBP (mmHg)	124.01 ± 16.25	121.87 ± 14.76	126.23 ± 17.54	1.02 (0.99–1.04)	0.171
DBP (mmHg)	76.94 ± 9.37	76.11 ± 7.47	77.81 ± 11.01	1.02 (0.98–1.06)	0.352
**Cancer type**				0.80 (0.46–1.38)	0.414
Lung cancer	62 (58.5)	29 (53.7)	33 (63.4)	1.50 (0.69–3.26)	0.309
Esophageal cancer	31 (29.2)	18 (33.3)	13 (25.0)	0.67 (0.29–1.55)	0.347
Other cancer	13 (12.3)	7 (13.0)	6 (5.7)	2.62 (0.75–9.10)	0.130
Surgorn	59 (55.7)	31 (57.4)	28 (53.8)	0.87 (0.40–1.86)	0.712
**Cancer stage**				0.96 (0.60–1.55)	0.873
Stage 1	4 (3.8)	1 (1.9)	3 (5.8)	3.25 (0.33–32.2)	0.315
Stage 2	32 (30.2)	17 (31.5)	15 (28.8)	0.88 (0.39–2.03)	0.768
Stage 3	47 (44.3)	25 (46.3)	22 (42.3)	0.85 (0.40–1.83)	0.680
Stage 4	23 (21.7)	11 (20.4)	12 (23.1)	1.17 (0.47–2.96)	0.736
**Cardiovascular disease and its risk factors**					
Smoking	56 (52.8)	30 (55.6)	26 (50.0)	0.80 (0.37–1.72)	0.567
Drinking	34 (32.1)	20 (37.0)	14 (26.9)	0.63 (0.27–1.43)	0.266
Hypertension	17 (16.0)	4 (7.4)	13 (25.0)	4.17 (1.26–13.78)	0.019
Diabetes	3 (2.8)	2 (3.7)	1 (1.9)	0.51 (0.05–5.80)	0.587
CHD	3 (2.8)	3 (5.6)	0	–	–
**Cardiac biomarker**					
CK-MB	1.002 ± 0.294	1.045 ± 0.640	0.958 ± 0.640	0.80 (0.43–1.51)	0.494
cTnT (μg/L)	0.0083 ± 0.0041	0.0086 ± 0.0041	0.0081 ± 0.0040	0.00 (0.00–1430)	0.483
NT-proBNP (ng/L)	72.24 ± 63.91	82.93 ± 79.84	61.55 ± 40.75	0.99 (0.99–1.00)	0.148
**Structure and function of left ventricle**					
LVEDd (mm)	46.05 ± 3.75	46.19 ± 3.37	45.94 ± 4.09	0.98 (0.88–1.10)	0.751
IVS (mm)	10.140 ± 1.07	10.02 ± 1.080	10.24 ± 1.06	1.21 (0.82–1.79)	0.329
LVPW (mm)	9.76 ± 1.04	9.67 ± 1.04	9.84 ± 1.04	1.17 (0.79–1.74)	0.441
LA (mm)	29.98 ± 3.40	29.28 ± 3.05	30.58 ± 3.60	1.13 (0.99–1.28)	0.070
LVEF (%)	62.75 ± 4.13	64.16 ± 3.68	61.54 ± 4.15	0.85 (0.76–0.95)	0.003
LVFS (%)	34.32 ± 2.83	34.95 ± 2.84	33.78 ± 2.73	0.86 (0.74–1.00)	0.048
E (cm/s)	79.79 ± 18.93	77.93 ± 16.59	81.46 ± 20.84	1.01 (0.99–1.03)	0.382
e’ (cm/s)	7.30 ± 1.39	7.64 ± 1.39	6.99 ± 1.33	0.69 (0.50–0.96)	0.029
E/e’	11.20 ± 2.94	10.43 ± 2.52	11.89 ± 3.15	1.22 (1.03–1.45)	0.024

LV, left ventricle; BMI, body mass index; SBP, systolic blood pressure; DBP, diastolic blood pressure; CHD, coronary heart disease; CK-MB, creatine kinase isoenzyme-MB; cTnT, cardiac troponin T; NT-proBNP, N-terminal pro brain natriuretic peptide; LVEDd, left ventricular internal dimension diastole; IVS, interventricular septal thickness diastolic; LVPW, left ventricular posterior wall; LA, left atrial diameter; LVEF, left ventricular ejection fraction; LVFS, left ventricular fraction shortening; E, peak early diastolic mitral inflow velocity; e’, peak early diastolic mitral annulus velocity; E/e’, the ratio of E to e’; OR, odd ratio; CI, confidence interval.

### Cancer types and treatment drugs

Of the 106 cancer patients treated with ICI, 62 (58.5%) were diagnosed with lung cancer, 31 (29.2%) with esophageal cancer, and 13 (12.3%) with other cancers. There were 4 (3.8%) patients in stage 1, 32 (30.2%) in stage 2, 47 (44.3%) in stage 3, 23 (21.7%) in stage 4, and 58 patients underwent surgery. The most commonly used ICI include carrelizumab, pembrolizumab, and tislelizumab (Table 2). In addition to ICI, most patients received conventional chemotherapy, the most common of which were docetaxel and albumin-paclitaxel (Table 3).

**TABLE 2:** The most commonly used immune checkpoint inhibitors (ICI) and their doses and frequencies.

Drug	Drug target	Approval agency	The usual dose and cycle of the drug		
Camrelizumab	PD-1	NMPA	200 mg	IV	Q3W
Tislelizumab	PD-1	NMPA	3 mg/kg	IV	Q3W
Pembrolizumab	PD-1	FDA, NMPA	2 mg/kg	IV	Q3W
Toripalimab	PD-1	NMPA	3 mg/kg	IV	Q3W
Durvalumab	PD-L1	FDA, NMPA	10 mg/kg	IV	Q3W
Navulumab	PD-1	FDA, NMPA	3 mg/kg	IV	Q3W
Sintilimab	PD-1	NMPA	200 mg	IV	Q3W

PD-1, programmed cell death 1; PD-L1, programmed cell death ligand 1; FDA, food and drug administration; NMPA, national medical products administration.

**TABLE 3:** Traditional chemotherapeutic drugs and their commonly used methods.

Drug	The usual dose and cycle of the drug		
Docetaxel	75 mg/m^2^	IV	Q3W
Albumin paclitaxel	260 mg/m^2^	IV	Q3W
Nedaplatin	75 mg/m^2^	IV	Q3W
Etoposide	150 mg (D1–D3)	IV	Q3W
Gemcitabine	1,250 mg/m^2^	IV	Q3W
Paclitaxel	100 mg/m^2^	IV	Q3W

### History of cardiovascular disease and its risk factors

Seventeen patients (16.0%) had a history of hypertension. There were 3 patients with coronary heart disease and 3 patients with diabetes (2.8% each). There were 56 cases (52.8%) with long-term smoking history and 34 cases (32.1%) with long-term drinking history (Table 1).

### ICI-related adverse cardiovascular events

**FIGURE 1:**
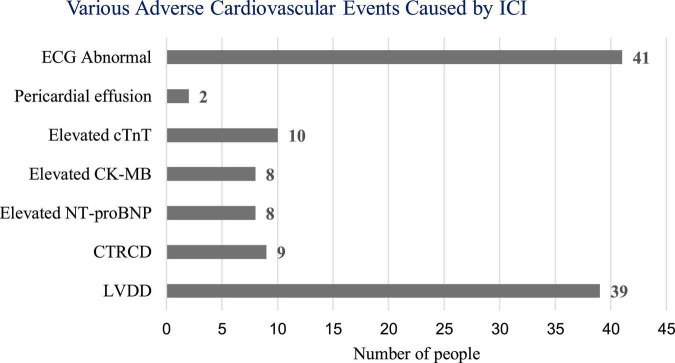
Number of adverse cardiovascular events caused by immune checkpoint inhibitors (ICI). ECG, electrocardiogram; cTnT, cardiac troponin T; CK-MB, creatine kinase isoenzyme-MB; NT-proBNP, N-terminal pro brain natriuretic peptide; CTRCD, cancer therapy-related cardiac dysfunction; LVDD, LV diastolic dysfunction.

In this study, ECG, cardiac biomarkers, echocardiography, etc., were used to detect ICI-related ACEs. Among the 106 patients, one patient (0.94%) developed ICI-associated myocarditis and died during hospitalization. Forty-one patients (38.68%) had new-onset ECG abnormalities, 39 patients (36.79%) had LVDD, and 9 patients (8.49%) had CTRCD. Two patients (1.89%) developed new pericardial effusion. Some patients had elevated cardiac biomarkers, including 10 patients (9.43%) with elevated cTnT, 8 patients (7.55%) with elevated creatine kinase isoenzyme-MB (CK-MB), and 8 patients with elevated NT-proBNP (7.55%) (Figure 1).

### ICI-associated myocarditis

**FIGURE 2:**
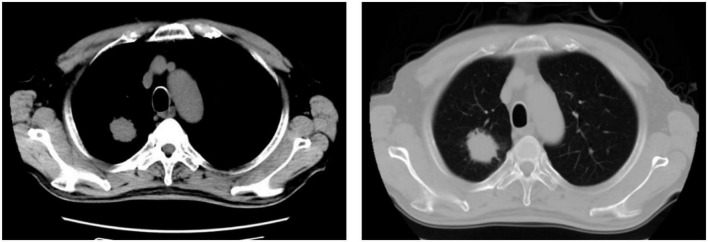
Chest computer tomography (CT) showed a space-occupying lesion in the right lung.

One patient developed ICI- associated myocarditis after receiving ICI. The patient is a 70-year-old man with right lung adenocarcinoma with bone metastases (Figure 2). Tumor stage was T2aN0M1cIVB. He had a history of hypertension (grade 3) for 6 years, and no history of diabetes or coronary heart disease. During the first hospitalization, cTnT, NT-proBNP, ECG, and echocardiogram were all normal. The first cycle was treated with 800 mg of pemetrexed and 130 mg of nedaplatin. Three weeks later, he was re-hospitalized for a second cycle of oncology treatment with pemetrexed 800 mg, nedaplatin 130 mg, and camrelizumab (a monoclonal antibody against PD-1) 200 mg. On day 20 after receiving camrelizumab, the patient developed shortness of breath. His self-measured heart rate was 34 beats per minute, and he had no chest pain, amaurosis, or syncope. He went to a nearby hospital for an ECG examination, which showed a third degree atrioventricular block. Laboratory tests showed CK-MB 31.5 ng/ml, myoglobin 1,103 ng/ml, cTnT 5.8 ng/ml, and NT-proBNP 1,280 ng/L. He was diagnosed with coronary heart disease and received related treatment, but his symptoms did not improve after 3 days.

**FIGURE 3:**
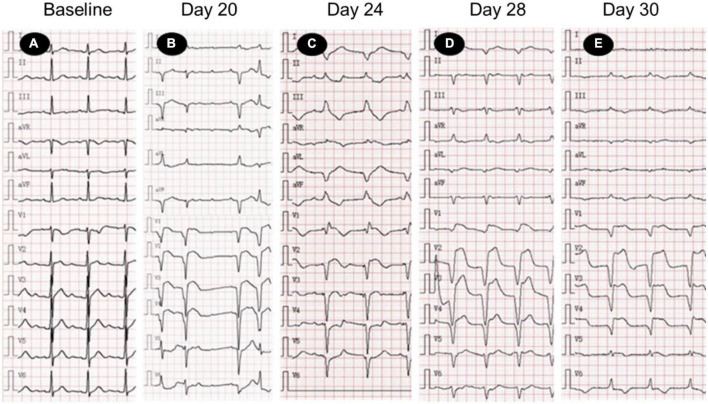
Electrocardiogram (ECG) changes in patients with acute myocarditis before and after immune checkpoint inhibitors (ICI) treatment. **(A)** Normal ECG; **(B)** sinus tachycardia, 3-degree atrioventricular block, frequent ventricular premature beats; **(C)** ventricular pacing rhythm (VVI, heart rate 70 times/min), ST segment elevation of anterior septum; **(D,E)** junctional tachycardia, ST segment elevation of anterior septum. ECG, electrocardiogram.

**FIGURE 4:**
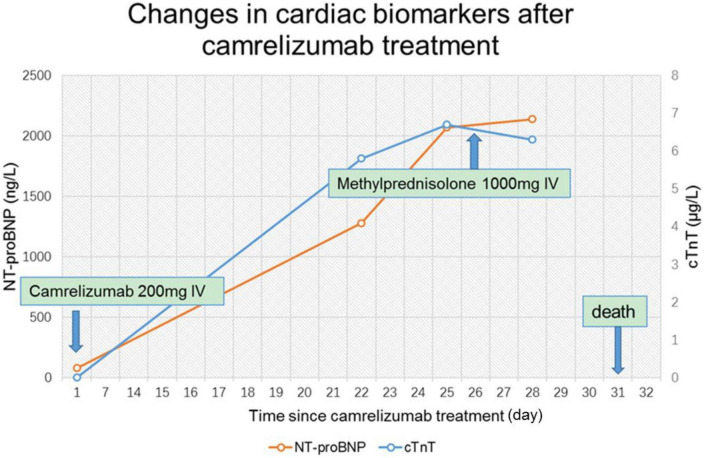
Changes of cardiac biomarkers in patients with acute myocarditis before and after immune checkpoint inhibitors (ICI) treatment. cTnT, cardiac troponin T; NT-proBNP, N-terminal pro brain natriuretic peptide.

On day 24 after receiving camrelizumab, he was admitted to the department of cardiovascular medicine of our hospital, and the ECG showed: (1) Sinus tachycardia; (2) Third-degree atrioventricular block with frequent accelerated idioventricular rhythm, ventricular escape; (3) Frequent multifocal premature ventricular contractions and short paroxysmal ventricular tachycardia; (4) Damaged ST-segment elevation in anterior wall and right ventricular leads. Echocardiography showed enlargement of right atrium and right ventricle; hypokinesis of right ventricular wall with decreased function; uncoordinated motion of LV wall and decreased LV function; moderate tricuspid regurgitation. Laboratory tests showed higher cardiac biomarkers than before. After discussion with the oncologist, the diagnosis of ICI-associated myocarditis was made. A temporary pacemaker was installed, and the ECG showed ventricular pacing rhythm. On the 26th day, intravenous infusion of methylprednisolone 1 g/d was given, but the patient gradually developed dyspnea, heart function got worse. The ECG on day 30 showed complete atrioventricular disjointness, junctional tachycardia, and anterior myocardial injury (Figure 3). The patient developed cardiac arrest in the early morning of the 31st day after receiving ICI, ECG monitoring showed pacing signal but no QRS complex and heartbeat, death still occurred after cardiopulmonary resuscitation (Figure 4).

### Abnormal ECG

A total of 41 patients (38.68%) had various new-onset ECG abnormalities, which were the most frequent cardiovascular events caused by ICI. Including ST-T changes, sinus bradycardia, sinus tachycardia, atrial premature beats, ventricular premature beats, bundle branch block, and atrioventricular block.

### Left ventricular dysfunction

The structure and function of the LV in patients undergoing ICI were examined by echocardiography. The results showed that the LV was enlarged in 2 cases (1.89%), and the left atrium (LA) was enlarged in 23 cases (21.70%). According to the diagnostic criteria, a total of 39 patients (36.79%) developed new-onset LVDD, and 9 patients (8.49%) developed new-onset CTRCD.

**FIGURE 5:**
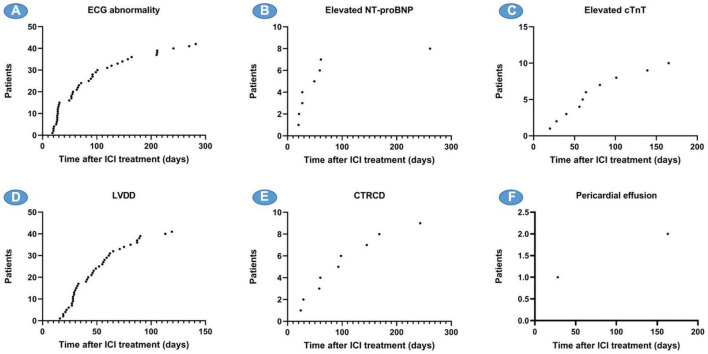
Time of onset of various ACEs since receiving ICI. **(A)** The number of patients with ECG abnormalities and the time of onset, **(B)** the number of patients with elevated NT-proBNP and the time of onset, **(C)** the number of patients with elevated cTnT and the time of onset, **(D)** the number of patients with LVDD and the time of onset, **(E)** the number of patients with CTRCD and the time of onset, and **(F)** the number of patients with pericardial effusion and the time of onset. ACEs, adverse cardiovascular events; NT-proBNP, N-terminal pro brain natriuretic peptide; cTnT, cardiac troponin T; LVDD, LV diastolic dysfunction; CTRCD, cancer therapy-related cardiac dysfunction.

Statistical analysis showed that the mean LVEF and mean LVFS after ICI treatment decreased from baseline (*P* < 0.001), while mean LA diameter increased (*P* < 0.001). LV end-diastolic diameter was not significantly different from baseline after ICI treatment (*P* = 0.473) (Table 4, Figure 5). In addition, the mean E/e’ increased compared to baseline (*P* = 0.001), and the mean e’ was significantly lower than baseline (*P* < 0.001) (Table 5, Figure 5).

**TABLE 4:** Mean LVEDd, LA, LVEF, and LVFS before and after immune checkpoint inhibitors (ICI) treatment.

Time (month)	*N*	LVEDd (cm)	LA (cm)	LVEF (%)	LVFS (%)
1	93	46.05 ± 3.75	29.98 ± 3.40	62.75 ± 4.13	34.32 ± 2.83
2	90	45.63 ± 3.94	31.47 ± 3.18*	59.30 ± 4.46*	33.07 ± 2.73*
3	58	45.78 ± 4.04	32.53 ± 3.05*	57.40 ± 4.29*	32.33 ± 2.73*
4	42	44.55 ± 3.98	31.71 ± 3.61*	57.95 ± 4.87*	31.81 ± 2.83*
5	32	45.66 ± 4.55	32.75 ± 3.37*	55.72 ± 4.10*	30.63 ± 2.39*
6	16	47.00 ± 5.39	32.44 ± 3.93*	55.93 ± 4.46*	30.34 ± 2.09*
7∼	15	45.40 ± 4.15	31.00 ± 3.96	57.53 ± 5.13*	31.47 ± 2.50*
Total	346	45.69 ± 4.06	31.42 ± 3.48*	59.18 ± 4.99*	32.71 ± 2.96*

*Compared with baseline, the difference was statistically significant (*P* < 0.05). LVEDd, left ventricular internal dimension diastole; IVS, interventricular septal thickness diastolic; LVPW, left ventricular posterior wall; LA, left atrial diameter; LVEF, left ventricular ejection fraction; LVFS, left ventricular fraction shortening.

**TABLE 5:** The average value of the indexes of left ventricle (LV) diastolic function before and after receiving immune checkpoint inhibitors (ICI).

Time (month)	*N*	E (cm)	e’ (cm)	E/e’
1	89	79.81 ± 18.95	7.29 ± 1.39	11.21 ± 2.96
2	75	79.99 ± 18.69	6.11 ± 1.26*	13.50 ± 4.12*
3	42	78.36 ± 14.02	6.05 ± 1.28*	13.33 ± 3.04*
4	35	75.34 ± 17.17	6.11 ± 1.35*	12.71 ± 3.25*
5	22	74.50 ± 15.09	6.23 ± 1.60*	12.45 ± 3.08
6	9	75.47 ± 15.04	5.88 ± 1.24*	13.28 ± 3.51
7∼	8	69.98 ± 21.89	5.66 ± 2.51*	15.01 ± 7.87*
Total	280	78.24 ± 17.67	6.46 ± 1.49	12.60 ± 3.69

*Compared with baseline, the difference was statistically significant (*P* < 0.05). LV, left ventricle; E, peak early diastolic mitral inflow velocity; e’, peak early diastolic mitral annulus velocity; E/e’, the ratio of E to e’.

### Time to adverse cardiovascular events

**FIGURE 6:**
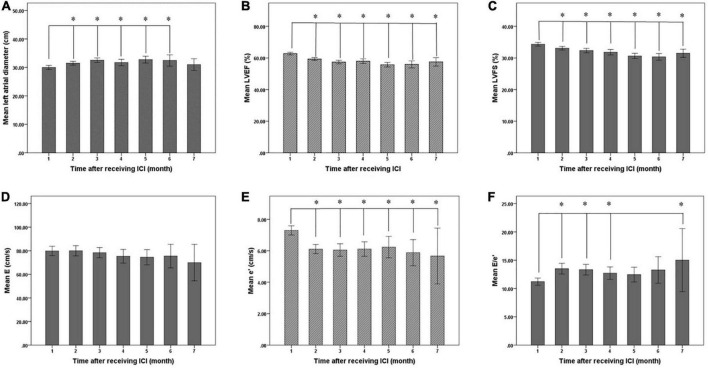
Changes of left ventricular systolic and diastolic function before (1 month) and after immune checkpoint inhibitors (ICI) treatment. **(A)** Mean left atrial diameter of patients before and after ICI treatment, **(B)** mean LVEF of patients before and after ICI treatment, **(C)** mean LVFS of patients before and after ICI treatment, **(D)** mean E of patients before and after ICI treatment, **(E)** mean e’ of patients before and after ICI treatment, and **(F)** mean E/e’ of patients before and after ICI treatment. LVEF, left ventricular ejection fraction; LVFS, left ventricular fraction shortening; E, peak early diastolic mitral inflow velocity; e’, peak early diastolic mitral annulus velocity; E/e’, the ratio of E to e’. *Compared with baseline, the difference was statistically significant (*P* < 0.05).

Electrocardiography (ECG) abnormalities and LVDD are the two most common cardiovascular system abnormalities caused by ICI, which began to appear 2–3 weeks after ICI treatment, and new cases continued to appear throughout the follow-up period, with an average onset time of 89.3 and 49.5 days, respectively. The elevation of NT-proBNP occurred more frequently in the first 10 weeks, while CTRCD and the elevation of cTnT occurred continuously during the follow-up period after receiving ICI, with an average onset time of 102 and 75.4 days, respectively (Figure 6).

### Risk factors of LVD caused by ICIs

**FIGURE 7:**
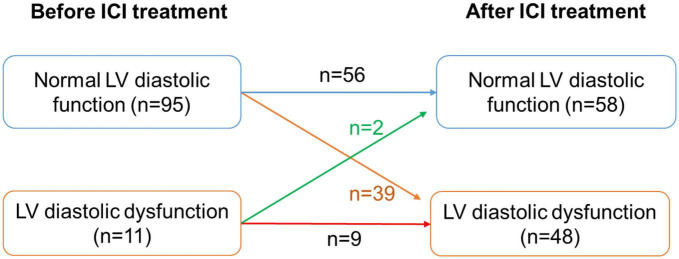
Number of patients with LV diastolic dysfunction before and after ICI treatment. LV, left ventricle.

Of the 106 patients treated with ICI, 52 developed LVD, including CTRCD or LVDD. Among them, before receiving ICI, the LVEF of all patients was above 50%, and 11 patients had LVDD. After ICI treatment, LV diastolic function returned to normal in 2 patients with LVDD, while 39 patients developed new-onset LVDD and 9 patients developed new-onset CTRCD (Figure 7). Risk factors for developing LVD were assessed using univariate regression analysis. Thirteen of the 52 patients with LVD had hypertension, while 4 of the 54 patients without LVD had hypertension. There was a significant difference between the two groups (OR = 4.17, 95% CI: 1.26–13.78; *P* = 0.019). Besides, the baseline LVEF and LVFS of patients with LVD were 61.54 ± 4.15% and 33.78 ± 2.73%, while those of the control group were 64.16 ± 3.68% and 34.95 ± 2.84, respectively. There was a statistically significant difference between the two groups (*P* = 0.003 and *P* = 0.048). Compared with patients without LVD, patients with LVD had lower e’ (6.99 ± 1.33 cm/s vs. 7.64 ± 1.39 cm/s, *P* = 0.029) and higher E to e’ ratio (11.89 ± 3.15 cm/s vs. 10.43 ± 2.52, *P* = 0.024). Baseline cardiac biomarkers, including CK-MB, cTnT, and NT-proBNP, were not significantly different between the two groups (Table 1).

Multivariate regression analysis showed that a history of hypertension (OR = 26.52, 95% CI: 2.479–283.667, *P* = 0.007) and lower baseline e’ (OR = 0.04, 95% CI: 0.003–0.709, *P* = 0.028) were risk factors for developing LVD (Table 6).

**TABLE 6:** Multivariate regression analysis with LVD.

Parameter	HR	95% CI	*P*-value
Hypertension	26.52	2.479–283.667	0.007
SBP (mmHg)	1.07	0.992–1.144	0.081
NT-proBNP (ng/L)	0.99	0.984–1.002	0.124
LA (mm)	1.08	0.804–1.458	0.602
LVEF (%)	0.80	0.629–1.008	0.058
LVFS (%)	0.59	0.324–1.075	0.085
e’ (cm/s)	0.04	0.003 - 0.709	0.028
E/e’	0.609	0.232 – 1.621	0.324

LVD, left ventricular dysfunction; SBP, systolic blood pressure; DBP, diastolic blood pressure; NT-proBNP, N-terminal pro brain natriuretic peptide; LA, left atrial diameter; LVEF, left ventricular ejection fraction; LVFS, left ventricular fraction shortening; e’, peak early diastolic mitral annulus velocity; E/e’, the ratio of E to e’; HR, hazard ratio; CI, confidence interval.

## Discussion

The incidence of various ACEs attributable to ICI may be underestimated, and it would be interesting to conduct studies to reveal their true incidence. We conducted this prospective clinical study to detect ICI-related cardiotoxicity. The main results are as follows: first, ICI can lead to various ACEs, such as acute myocarditis, various ECG abnormalities, LVD including CTRCD and LVDD, and pericardial effusion. Its incidence is higher than that reported in many previous articles. Second, echocardiography is a useful method to detect LV systolic and diastolic dysfunction caused by ICI, and a higher incidence of CTRCD and LVDD has been found. Third, hypertension and poor baseline LV systolic or diastolic function are predictors of LVD including CTRCD and LVDD. Last, combining echocardiography, ECG, cardiac biomarkers, chest CT, etc., can early detect cardiotoxicity caused by ICI, so that patients can receive timely diagnosis and treatment, thereby improving the prognosis.

Immune checkpoint inhibitors (ICI) can lead to acute myocarditis, with an incidence of 0.27 to 1.14% (18, 19), most of which occurs about 1 month after receiving ICI (20). One case of acute myocarditis occurred in the patients included in this study. The earliest clinical symptoms were dyspnea, and the ECG showed complete atrioventricular block. Abnormal findings on cardiac biomarkers and echocardiography further confirmed the diagnosis of myocarditis. Although the patient has been implanted with a pacemaker, ICI has led to myocardial injury, edema and heart failure and the patient may eventually die of heart failure and cardiac electromechanical separation. It is very important to closely observe whether there are cardiovascular symptoms such as dyspnea in patients receiving ICI. Once symptoms are present, early examination including ECG, cardiac biomarkers, echocardiography, or cardiac magnetic resonance (CMR) is helpful in diagnosing acute myocarditis. If possible, myocardial histopathology should be performed to confirm the diagnosis, otherwise, CMR is the first choice for non-invasive diagnosis of myocarditis (21).

With the extensive clinical application of ICI, cases of ICI-related myocarditis have been reported continuously. Due to its high risk and high mortality, it has attracted more and more attention from oncologists and cardiologists (22, 23). However, the current understanding of cardiac dysfunction caused by ICI, especially chronic LV systolic and diastolic dysfunction, is still insufficient, and there are few reports. 2022 ESC Guidelines on cardio oncology mentioned that ICI can lead to asymptomatic or symptomatic CTRCD, which is one of the clinical manifestations of cardiovascular toxicity of ICI, indicating that cardiac dysfunction caused by ICI has begun to attract attention in the field of oncocardiology (24). The CTRCD caused by ICI is also called non-inflammatory left ventricular dysfunction. A retrospective study showed that the median time from the beginning of ICI treatment to presentation with myocarditis was 12 weeks, while the time to presentation with non-inflammatory left ventricular dysfunction was 26 weeks (25). This study found a higher incidence of LVD caused by ICI, including CTRCD and LVDD, which was only lower than ECG abnormalities. These results suggest that regular assessment of cardiac function during ICI therapy can aid in the early detection of LVD, which may help some patients receive timely treatment to improve outcomes.

Non-invasive cardiac function evaluation methods mainly include CMR and echocardiography. CMR is still the current “gold standard” for non-invasive evaluation of cardiac structure and function. Moreover, with the development in recent years, the ability of CMR to assess myocardial fibrosis (MF) has been confirmed by studies, and MF is closely related to the prognosis of cardiovascular disease (26). However, CMR is expensive, requires injection of contrast agent, takes a long time, and has contraindications for examination, making it difficult to be used as a routine clinical examination method.

Echocardiography is the most common method for non-invasive visualization of cardiac structure and assessment of cardiac function. It has the advantages of convenient operation, no contraindications, low price, non-invasive and non-radioactive. At present, echocardiography has been widely used in the evaluation of cardiac function before, during and after cancer therapy. However, research on ICI-induced LVD, especially LVDD, is still lacking. In this study, echocardiography was used to evaluate cardiac function in cancer patients treated with ICI. LV systolic dysfunction is one of the most common ACEs in cancer treatment and is defined as CTRCD. According to the latest recommendations from the British Society of Echocardiography (BSE) and the British Society of Cardio-Oncology (BCOS), a decline in LVEF of > 10 absolute percentage points to a value of < 50% is defined as CTRCD (17). According to this definition, 9 patients developed CTRCD after ICI treatment, an incidence of 8.49%. This incidence is higher than some database-based reports and may be related to the fact that most patients treated with ICI did not undergo routine echocardiography, leading to an underestimation of the incidence of CTRCD.

Heart failure with preserved ejection fraction (HFpEF) has received increasing attention in recent years, its prevalence is high, accounting for 50% of all HF, and it is associated with significant morbidity and mortality (27, 28). Asymptomatic LVDD has been shown to be an independent predictor of late progression to HFpEF (29, 30), of which 13–28% may later develop HFpEF, so early diagnosis and intervention of LVDD may improve patient outcomes.

In this study, echocardiography was used to assess LV diastolic function. The results suggest that ICI can lead to a high incidence of LVDD. A total of 39 patients developed new-onset LVDD, and the incidence was 36.79%, which was significantly higher than that of CTRCD. It may be a more sensitive indicator for early detection of LVD and ICI-related cardiotoxicity. In addition, mean e’ and E/e’ were significantly higher than baseline after patients received ICI, suggesting that ICI resulted in a decrease in overall LV diastolic function. These results suggest that regular echocardiography can early detect LVD caused by ICI, including CTRCD and LVDD.

Myocardial fibrosis is one of the main pathological bases of arrhythmia and heart failure in many heart diseases. A meta-analysis showed that among patients with ICI associated myocarditis, CMR showed a prevalence of edema of 9 to 60% and a prevalence of late gadolinium enhancement (LGE) of 23 to 83% (31). This result suggested that ICI can lead to myocardial fibrosis, which may be the pathological basis of cardiac dysfunction in many patients.

Electrocardiography (ECG) can be used to detect cardiotoxicity associated with tumor therapy, which is convenient, fast and non-invasive. It can be used for early detection of arrhythmia and myocardial injury caused by tumor drugs (32). This study found that after ICI treatment, a total of 39 patients developed various new ECG abnormalities, including ST depression, T-wave inversions, sinus bradycardia, sinus tachycardia, premature atrial contractions, premature ventricular contractions, bundle branch block, and atrioventricular block. Its incidence is highest among ICI-related ACEs, suggesting that ECG abnormalities are sensitive markers for early assessment of ICI-related cardiotoxicity. Although ECG abnormalities are not specific and may occur in patients with subclinical myocardial injury and severe myocarditis, new ECG abnormalities may indicate the occurrence of cardiotoxicity. As previously described, this patient with ICI-associated myocarditis developed new ECG abnormalities, including atrioventricular block, ST-segment depression, and T-wave inversion. This shows that patients with abnormal ECG need to be concerned and followed up.

Immune myocarditis and heart failure are major causes of ICI-related death. Cardiac biomarkers are useful indicators of myocardial inflammation, injury and cardiac dysfunction (33). LVD induced by oncology drugs is a gradual development process, which first causes inflammatory damage of cardiomyocytes, and then gradually develops cardiac dysfunction (18). Studies have shown that elevated cardiac biomarkers such as BNP/NT-proBNP, cardiac troponin are associated with subsequent development of CTRCD or LVDD (34). Furthermore, in some patients, abnormal cardiac biomarkers appeared earlier than the time when LVEF was below normal (35). Therefore, regular cardiac biomarker testing can aid in the early detection of cardiotoxicity, such as myocarditis, heart failure, and asymptomatic subclinical cardiac injury. In this study, after ICI treatment, 10 patients developed cTnT elevation, 8 patients developed CK-MB elevation, and 8 patients developed NT-proBNP elevation, which should be followed closely.

We analyzed the risk factors for LVD in the enrolled patients after receiving ICI. Univariate regression analysis showed that hypertension was an independent risk factor for LVD. This is consistent with hypertension being one of the major risk factors for the development of LVDD (36). Furthermore, lower baseline systolic and diastolic function was associated with higher subsequent LVD incidence. Specifically, patients in the LVD group had lower baseline LVEF, LVFS, and e’, and higher ratios of E to e’, compared with controls. Multivariate regression analysis also showed that hypertensive patients had a higher incidence of LVD, and poorer systolic and diastolic function at baseline was associated with a higher subsequent incidence of LVD.

## Study limitations

The current study has several limitations. First, it is a single-center prospective study. Second, the relatively small number of patients included in the study reduces the statistical power of our results. Especially when analyzing the risk factors for ICI leading to ACE, some possible risk factors may be missed. Third, in this study, patients underwent periodic echocardiography to assess their systolic and diastolic function. However, some patients developed cardiac displacement after extensive lobectomy, which resulted in unsuccessful measurement of global long-axis strain (GLS) of the LV. GLS may be a more sensitive measure of LV systolic function than LVEF. Last, because many patients treated with ICI have advanced cancer, follow-up time is inconsistent, and the long-term incidence of cardiotoxicity with ICI may be underestimated.

## Conclusion

Immune checkpoint inhibitors (ICI) can lead to a variety of ACEs, including acute myocarditis, pericardial effusion, LVD, ECG abnormalities, and cardiac biomarker abnormalities. Its incidence is higher than some previous retrospective studies or database-based reports. The combined use of ECG, cardiac biomarkers, echocardiography, and CMR is helpful to early identify the cardiovascular toxicity of ICI. LVD, including CTRCD and LVDD, has a high incidence, and regular echocardiography is helpful for early detection of LVD. Patients with hypertension or poor LV systolic or diastolic function at baseline were predictors of LVD after ICI.

## Data availability statement

The original contributions presented in this study are included in this article/supplementary material, further inquiries can be directed to the corresponding author.

## Ethics statement

The studies involving human participants were reviewed and approved by the Ethics Committee of the First Affiliated Hospital of Chongqing Medical University (ethics number, 2018-10-2). The patients/participants provided their written informed consent to participate in this study.

## Author contributions

CZ conducted this clinical study, statistical analysis, and manuscript writing. SQ provided the direction and guidance throughout the preparation of this manuscript. ZZ conceived the comment and revised the manuscript. ZLC and LJS assisted in the collection of clinical data. YXZ reviewed and edited the manuscript before submission. All authors read and approved the final manuscript.
